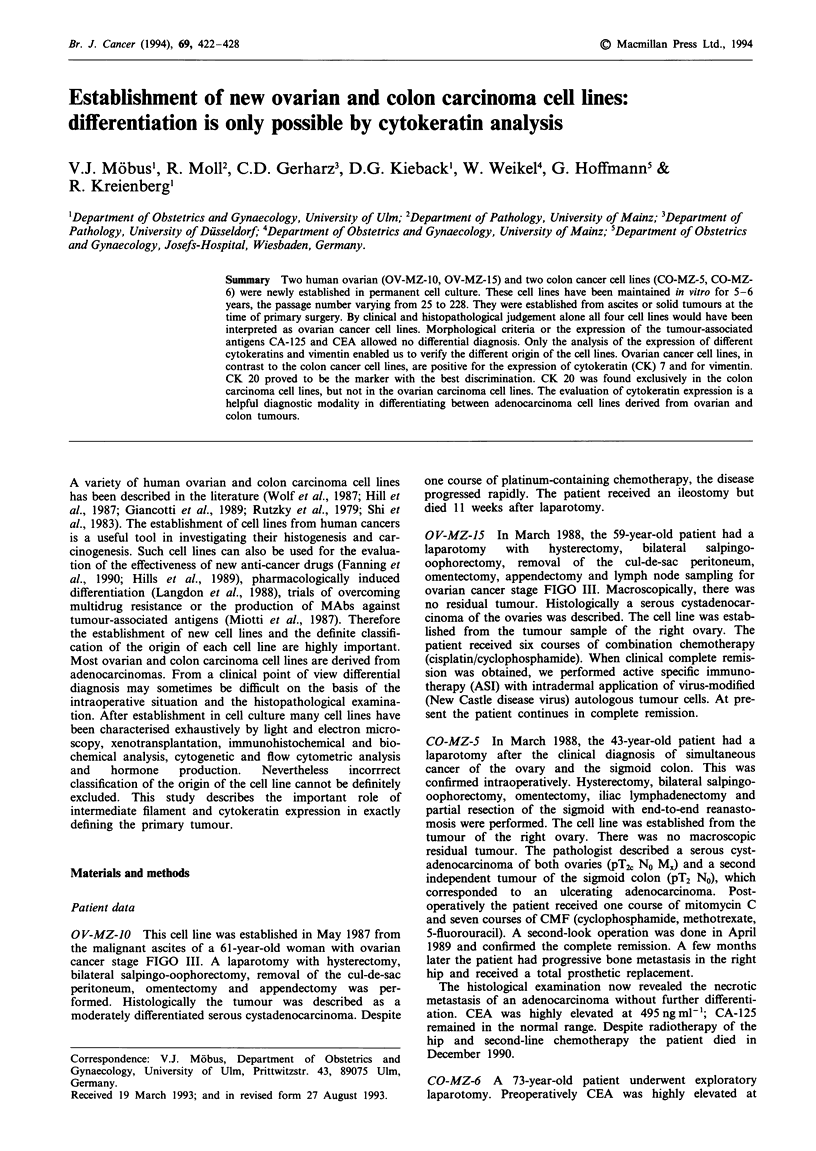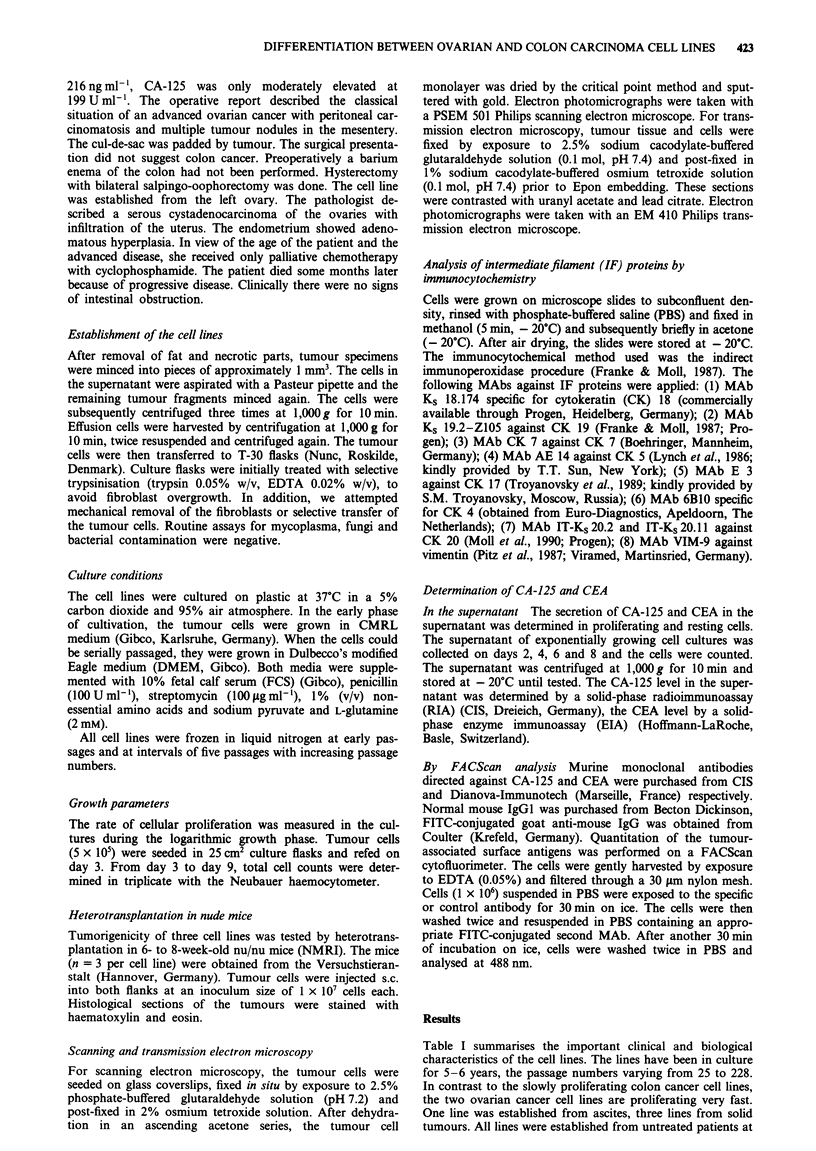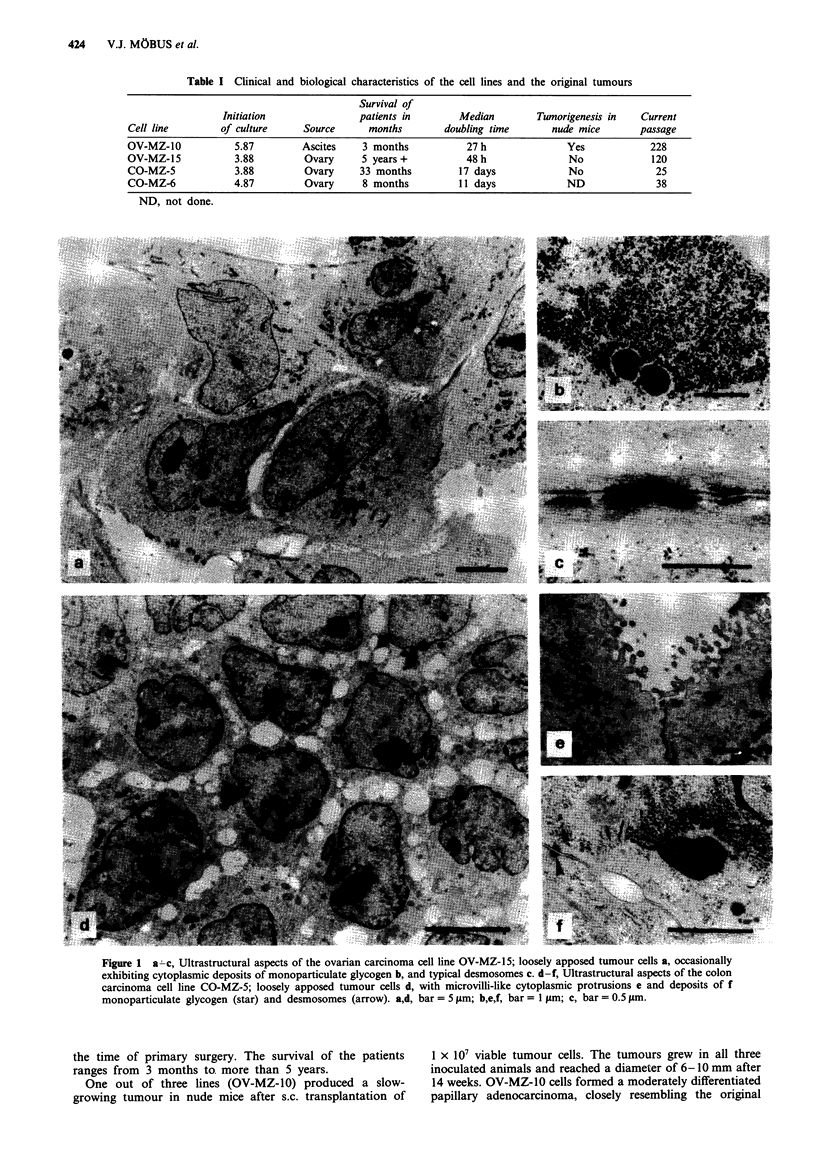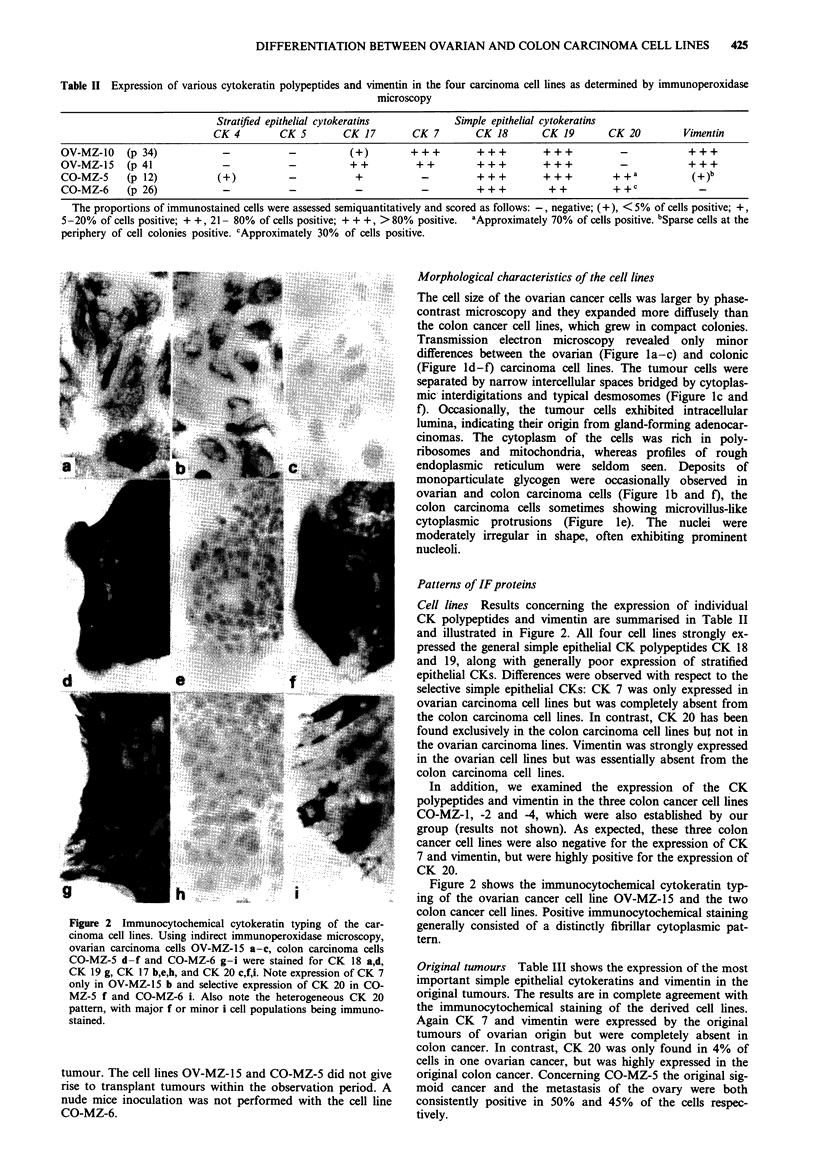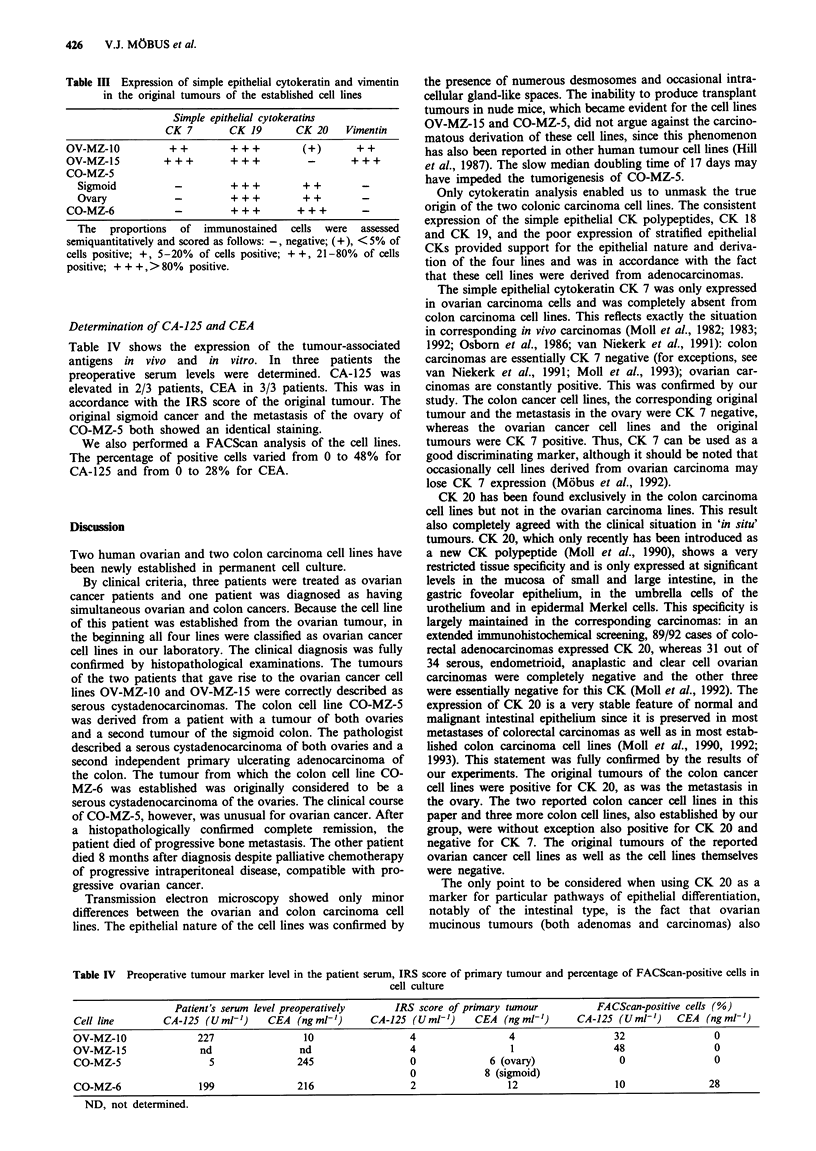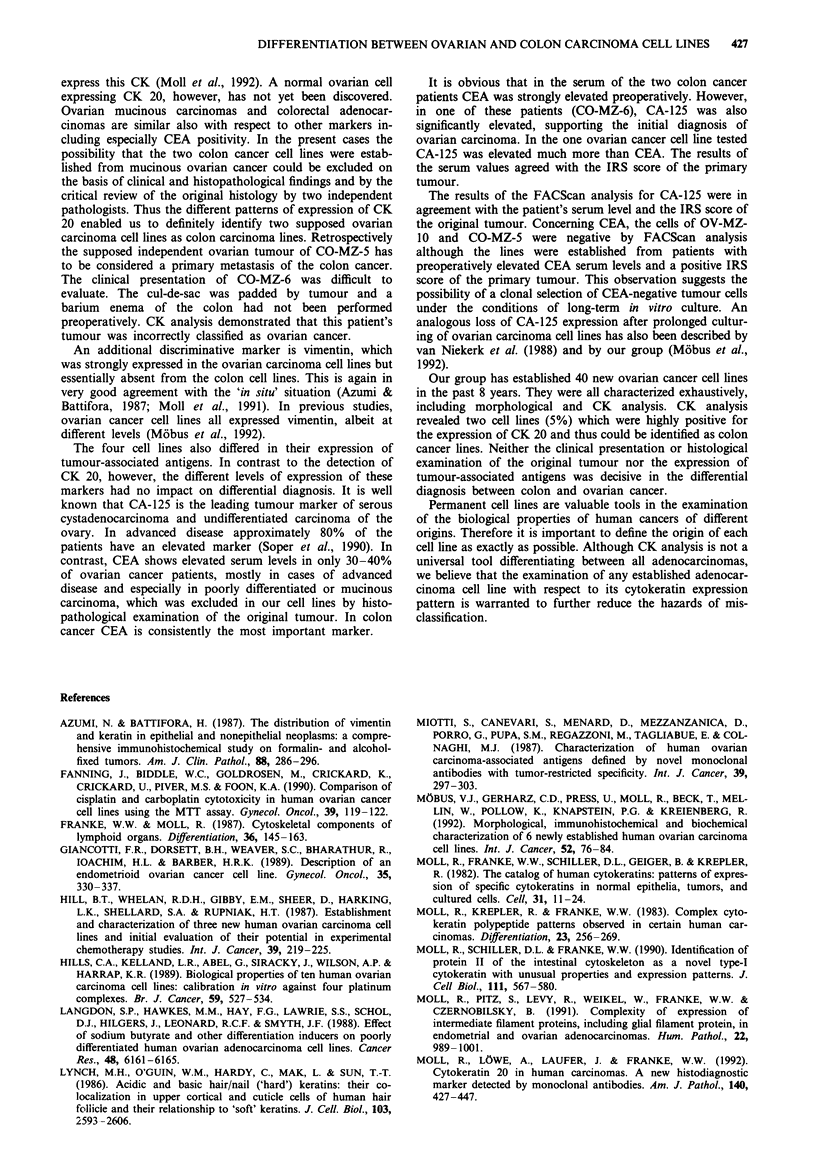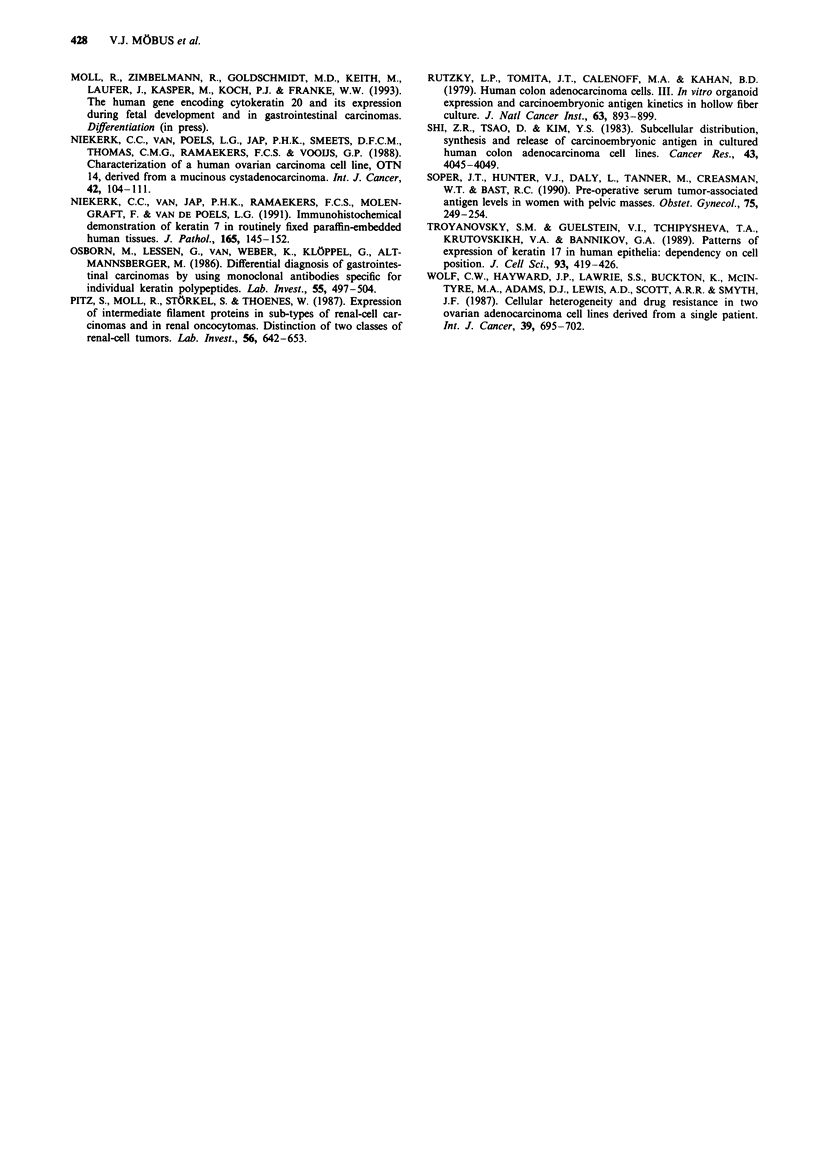# Establishment of new ovarian and colon carcinoma cell lines: differentiation is only possible by cytokeratin analysis.

**DOI:** 10.1038/bjc.1994.78

**Published:** 1994-03

**Authors:** V. J. Möbus, R. Moll, C. D. Gerharz, D. G. Kieback, W. Weikel, G. Hoffmann, R. Kreienberg

**Affiliations:** Department of Obstetrics and Gynaecology, University of Ulm, Germany.

## Abstract

**Images:**


Br. J. Cancer (1994), 69, 422-428 © Macmillan Press Ltd., 1994

### Establishment of new ovarian and colon carcinoma cell lines: differentiation is only possible by cytokeratin analysis

V.J. Möbus[1], R. Moll[2], C.D. Gerharz[3], D.G. Kieback[1], W. Weikel[4], G. Hoffmann[5] & R. Kreienberg[1]

[1]*Department of Obstetrics and Gynaecology, University of Ulm;* [2]*Department of Pathology, University of Mainz;* [3]*Department of Pathology, University of Düsseldorf;* [4]*Department of Obstetrics and Gynaecology, University of Mainz;* [5]*Department of Obstetrics and Gynaecology, Josefs-Hospital, Wiesbaden, Germany.*

**Summary** Two human ovarian (OV-MZ-10, OV-MZ-15) and two colon cancer cell lines (CO-MZ-5, CO-MZ-6) were newly established in permanent cell culture. These cell lines have been maintained *in vitro* for 5–6 years, the passage number varying from 25 to 228. They were established from ascites or solid tumours at the time of primary surgery. By clinical and histopathological judgement alone all four cell lines would have been interpreted as ovarian cancer cell lines. Morphological criteria or the expression of the tumour-associated antigens CA-125 and CEA allowed no differential diagnosis. Only the analysis of the expression of different cytokeratins and vimentin enabled us to verify the different origin of the cell lines. Ovarian cancer cell lines, in contrast to the colon cancer cell lines, are positive for the expression of cytokeratin (CK) 7 and for vimentin. CK 20 proved to be the marker with the best discrimination. CK 20 was found exclusively in the colon carcinoma cell lines, but not in the ovarian carcinoma cell lines. The evaluation of cytokeratin expression is a helpful diagnostic modality in differentiating between adenocarcinoma cell lines derived from ovarian and colon tumours.

A variety of human ovarian and colon carcinoma cell lines has been described in the literature (Wolf *et al.*, 1987; Hill *et al.*, 1987; Giancotti *et al.*, 1989; Rutzky *et al.*, 1979; Shi *et al.*, 1983). The establishment of cell lines from human cancers is a useful tool in investigating their histogenesis and carcinogenesis. Such cell lines can also be used for the evaluation of the effectiveness of new anti-cancer drugs (Fanning *et al.*, 1990; Hills *et al.*, 1989), pharmacologically induced differentiation (Langdon *et al.*, 1988), trials of overcoming multidrug resistance or the production of MAbs against tumour-associated antigens (Miotti *et al.*, 1987). Therefore the establishment of new cell lines and the definite classification of the origin of each cell line are highly important. Most ovarian and colon carcinoma cell lines are derived from adenocarcinomas. From a clinical point of view differential diagnosis may sometimes be difficult on the basis of the intraoperative situation and the histopathological examination. After establishment in cell culture many cell lines have been characterised exhaustively by light and electron microscopy, xenotransplantation, immunohistochemical and biochemical analysis, cytogenetic and flow cytometric analysis and hormone production. Nevertheless incorrrect classification of the origin of the cell line cannot be definitely excluded. This study describes the important role of intermediate filament and cytokeratin expression in exactly defining the primary tumour.

### Materials and methods

### *Patient data*

*OV-MZ-10* This cell line was established in May 1987 from the malignant ascites of a 61-year-old woman with ovarian cancer stage FIGO III. A laparotomy with hysterectomy, bilateral salpingo-oophorectomy, removal of the cul-de-sac peritoneum, omentectomy and appendectomy was performed. Histologically the tumour was described as a moderately differentiated serous cystadenocarcinoma. Despite one course of platinum-containing chemotherapy, the disease progressed rapidly. The patient received an ileostomy but died 11 weeks after laparotomy.

*OV-MZ-15* In March 1988, the 59-year-old patient had a laparotomy with hysterectomy, bilateral salpingo-oophorectomy, removal of the cul-de-sac peritoneum, omentectomy, appendectomy and lymph node sampling for ovarian cancer stage FIGO III. Macroscopically, there was no residual tumour. Histologically a serous cystadenocarcinoma of the ovaries was described. The cell line was established from the tumour sample of the right ovary. The patient received six courses of combination chemotherapy (cisplatin/cyclophosphamide). When clinical complete remission was obtained, we performed active specific immunotherapy (ASI) with intradermal application of virus-modified (New Castle disease virus) autologous tumour cells. At present the patient continues in complete remission.

*CO-MZ-5* In March 1988, the 43-year-old patient had a laparotomy after the clinical diagnosis of simultaneous cancer of the ovary and the sigmoid colon. This was confirmed intraoperatively. Hysterectomy, bilateral salpingo-oophorectomy, omentectomy, iliac lymphadenectomy and partial resection of the sigmoid with end-to-end reanastomosis were performed. The cell line was established from the tumour of the right ovary. There was no macroscopic residual tumour. The pathologist described a serous cystadenocarcinoma of both ovaries ($pT_{2c}$ $N_0$ $M_x$) and a second independent tumour of the sigmoid colon ($pT_2$ $N_0$), which corresponded to an ulcerating adenocarcinoma. Postoperatively the patient received one course of mitomycin C and seven courses of CMF (cyclophosphamide, methotrexate, 5-fluorouracil). A second-look operation was done in April 1989 and confirmed the complete remission. A few months later the patient had progressive bone metastasis in the right hip and received a total prosthetic replacement.

The histological examination now revealed the necrotic metastasis of an adenocarcinoma without further differentiation. CEA was highly elevated at 495 ng $ml^{-1}$; CA-125 remained in the normal range. Despite radiotherapy of the hip and second-line chemotherapy the patient died in December 1990.

*CO-MZ-6* A 73-year-old patient underwent exploratory laparotomy. Preoperatively CEA was highly elevated at

Correspondence: V.J. Möbus, Department of Obstetrics and Gynaecology, University of Ulm, Prittwitzstr. 43, 89075 Ulm, Germany.
Received 19 March 1993; and in revised form 27 August 1993.

216 ng $ml^{-1}$, CA-125 was only moderately elevated at 199 U $ml^{-1}$. The operative report described the classical situation of an advanced ovarian cancer with peritoneal carcinomatosis and multiple tumour nodules in the mesentery. The cul-de-sac was padded by tumour. The surgical presentation did not suggest colon cancer. Preoperatively a barium enema of the colon had not been performed. Hysterectomy with bilateral salpingo-oophorectomy was done. The cell line was established from the left ovary. The pathologist described a serous cystadenocarcinoma of the ovaries with infiltration of the uterus. The endometrium showed adenomatous hyperplasia. In view of the age of the patient and the advanced disease, she received only palliative chemotherapy with cyclophosphamide. The patient died some months later because of progressive disease. Clinically there were no signs of intestinal obstruction.

*Establishment of the cell lines*

After removal of fat and necrotic parts, tumour specimens were minced into pieces of approximately 1 $mm^3$. The cells in the supernatant were aspirated with a Pasteur pipette and the remaining tumour fragments minced again. The cells were subsequently centrifuged three times at 1,000 *g* for 10 min. Effusion cells were harvested by centrifugation at 1,000 g for 10 min, twice resuspended and centrifuged again. The tumour cells were then transferred to T-30 flasks (Nunc, Roskilde, Denmark). Culture flasks were initially treated with selective trypsinisation (trypsin 0.05% w/v, EDTA 0.02% w/v), to avoid fibroblast overgrowth. In addition, we attempted mechanical removal of the fibroblasts or selective transfer of the tumour cells. Routine assays for mycoplasma, fungi and bacterial contamination were negative.

*Culture conditions*

The cell lines were cultured on plastic at 37°C in a 5% carbon dioxide and 95% air atmosphere. In the early phase of cultivation, the tumour cells were grown in CMRL medium (Gibco, Karlsruhe, Germany). When the cells could be serially passaged, they were grown in Dulbecco's modified Eagle medium (DMEM, Gibco). Both media were supplemented with 10% fetal calf serum (FCS) (Gibco), penicillin (100 U $ml^{-1}$), streptomycin (100 $\mu g$ $ml^{-1}$), 1% (v/v) non-essential amino acids and sodium pyruvate and L-glutamine (2 mM).

All cell lines were frozen in liquid nitrogen at early passages and at intervals of five passages with increasing passage numbers.

*Growth parameters*

The rate of cellular proliferation was measured in the cultures during the logarithmic growth phase. Tumour cells ($5 \times 10^5$) were seeded in 25 $cm^2$ culture flasks and refed on day 3. From day 3 to day 9, total cell counts were determined in triplicate with the Neubauer haemocytometer.

*Heterotransplantation in nude mice*

Tumorigenicity of three cell lines was tested by heterotransplantation in 6- to 8-week-old nu/nu mice (NMRI). The mice ($n = 3$ per cell line) were obtained from the Versuchstieranstalt (Hannover, Germany). Tumour cells were injected s.c. into both flanks at an inoculum size of $1 \times 10^7$ cells each. Histological sections of the tumours were stained with haematoxylin and eosin.

*Scanning and transmission electron microscopy*

For scanning electron microscopy, the tumour cells were seeded on glass coverslips, fixed *in situ* by exposure to 2.5% phosphate-buffered glutaraldehyde solution (pH 7.2) and post-fixed in 2% osmium tetroxide solution. After dehydration in an ascending acetone series, the tumour cell monolayer was dried by the critical point method and sputtered with gold. Electron photomicrographs were taken with a PSEM 501 Philips scanning electron microscope. For transmission electron microscopy, tumour tissue and cells were fixed by exposure to 2.5% sodium cacodylate-buffered glutaraldehyde solution (0.1 mol, pH 7.4) and post-fixed in 1% sodium cacodylate-buffered osmium tetroxide solution (0.1 mol, pH 7.4) prior to Epon embedding. These sections were contrasted with uranyl acetate and lead citrate. Electron photomicrographs were taken with an EM 410 Philips transmission electron microscope.

*Analysis of intermediate filament (IF) proteins by immunocytochemistry*

Cells were grown on microscope slides to subconfluent density, rinsed with phosphate-buffered saline (PBS) and fixed in methanol (5 min, − 20°C) and subsequently briefly in acetone (− 20°C). After air drying, the slides were stored at − 20°C. The immunocytochemical method used was the indirect immunoperoxidase procedure (Franke & Moll, 1987). The following MAbs against IF proteins were applied: (1) MAb $K_S$ 18.174 specific for cytokeratin (CK) 18 (commercially available through Progen, Heidelberg, Germany); (2) MAb $K_S$ 19.2–Z105 against CK 19 (Franke & Moll, 1987; Progen); (3) MAb CK 7 against CK 7 (Boehringer, Mannheim, Germany); (4) MAb AE 14 against CK 5 (Lynch *et al.*, 1986; kindly provided by T.T. Sun, New York); (5) MAb E 3 against CK 17 (Troyanovsky *et al.*, 1989; kindly provided by S.M. Troyanovsky, Moscow, Russia); (6) MAb 6B10 specific for CK 4 (obtained from Euro-Diagnostics, Apeldoorn, The Netherlands); (7) MAb IT-$K_S$ 20.2 and IT-$K_S$ 20.11 against CK 20 (Moll *et al.*, 1990; Progen); (8) MAb VIM-9 against vimentin (Pitz *et al.*, 1987; Viramed, Martinsried, Germany).

*Determination of CA-125 and CEA*

*In the supernatant* The secretion of CA-125 and CEA in the supernatant was determined in proliferating and resting cells. The supernatant of exponentially growing cell cultures was collected on days 2, 4, 6 and 8 and the cells were counted. The supernatant was centrifuged at 1,000 *g* for 10 min and stored at − 20°C until tested. The CA-125 level in the supernatant was determined by a solid-phase radioimmunoassay (RIA) (CIS, Dreieich, Germany), the CEA level by a solid-phase enzyme immunoassay (EIA) (Hoffmann-LaRoche, Basle, Switzerland).

*By FACScan analysis* Murine monoclonal antibodies directed against CA-125 and CEA were purchased from CIS and Dianova-Immunotech (Marseille, France) respectively. Normal mouse IgG1 was purchased from Becton Dickinson, FITC-conjugated goat anti-mouse IgG was obtained from Coulter (Krefeld, Germany). Quantitation of the tumour-associated surface antigens was performed on a FACScan cytofluorimeter. The cells were gently harvested by exposure to EDTA (0.05%) and filtered through a 30 μm nylon mesh. Cells ($1 \times 10^6$) suspended in PBS were exposed to the specific or control antibody for 30 min on ice. The cells were then washed twice and resuspended in PBS containing an appropriate FITC-conjugated second MAb. After another 30 min of incubation on ice, cells were washed twice in PBS and analysed at 488 nm.

### Results

Table I summarises the important clinical and biological characteristics of the cell lines. The lines have been in culture for 5–6 years, the passage numbers varying from 25 to 228. In contrast to the slowly proliferating colon cancer cell lines, the two ovarian cancer cell lines are proliferating very fast. One line was established from ascites, three lines from solid tumours. All lines were established from untreated patients at

**Table I** Clinical and biological characteristics of the cell lines and the original tumours

| *Cell line* | *Initiation of culture* | *Source* | *Survival of patients in months* | *Median doubling time* | *Tumorigenesis in nude mice* | *Current passage* |
|---|---|---|---|---|---|---|
| OV-MZ-10 | 5.87 | Ascites | 3 months | 27 h | Yes | 228 |
| OV-MZ-15 | 3.88 | Ovary | 5 years + | 48 h | No | 120 |
| CO-MZ-5 | 3.88 | Ovary | 33 months | 17 days | No | 25 |
| CO-MZ-6 | 4.87 | Ovary | 8 months | 11 days | ND | 38 |

ND, not done.

**Figure 1** **a–c**, Ultrastructural aspects of the ovarian carcinoma cell line OV-MZ-15; loosely apposed tumour cells **a**, occasionally exhibiting cytoplasmic deposits of monoparticulate glycogen **b**, and typical desmosomes **c**. **d–f**, Ultrastructural aspects of the colon carcinoma cell line CO-MZ-5; loosely apposed tumour cells **d**, with microvilli-like cytoplasmic protrusions **e** and deposits of **f** monoparticulate glycogen (star) and desmosomes (arrow). **a**,**d**, bar = 5 μm; **b**,**e**,**f**, bar = 1 μm; **c**, bar = 0.5 μm.

the time of primary surgery. The survival of the patients ranges from 3 months to more than 5 years.

One out of three lines (OV-MZ-10) produced a slow-growing tumour in nude mice after s.c. transplantation of $1 \times 10^7$ viable tumour cells. The tumours grew in all three inoculated animals and reached a diameter of 6–10 mm after 14 weeks. OV-MZ-10 cells formed a moderately differentiated papillary adenocarcinoma, closely resembling the original

**Table II** Expression of various cytokeratin polypeptides and vimentin in the four carcinoma cell lines as determined by immunoperoxidase microscopy

| | | *Stratified epithelial cytokeratins* | | | *Simple epithelial cytokeratins* | | | | |
|---|---|---|---|---|---|---|---|---|---|
| | | *CK 4* | *CK 5* | *CK 17* | *CK 7* | *CK 18* | *CK 19* | *CK 20* | *Vimentin* |
| OV-MZ-10 | (p 34) | − | − | (+) | +++ | +++ | +++ | − | +++ |
| OV-MZ-15 | (p 41 | − | − | ++ | ++ | +++ | +++ | − | +++ |
| CO-MZ-5 | (p 12) | (+) | − | + | − | +++ | +++ | ++[a] | (+)[b] |
| CO-MZ-6 | (p 26) | − | − | − | − | +++ | ++ | ++[c] | − |

The proportions of immunostained cells were assessed semiquantitatively and scored as follows: −, negative; (+), <5% of cells positive; +, 5–20% of cells positive; ++, 21– 80% of cells positive; +++, >80% positive. [a]Approximately 70% of cells positive. [b]Sparse cells at the periphery of cell colonies positive. [c]Approximately 30% of cells positive.

**Figure 2** Immunocytochemical cytokeratin typing of the carcinoma cell lines. Using indirect immunoperoxidase microscopy, ovarian carcinoma cells OV-MZ-15 **a**–**c**, colon carcinoma cells CO-MZ-5 **d**–**f** and CO-MZ-6 **g**–**i** were stained for CK 18 **a**,**d**, CK 19 **g**, CK 17 **b**,**e**,**h**, and CK 20 **c**,**f**,**i**. Note expression of CK 7 only in OV-MZ-15 **b** and selective expression of CK 20 in CO-MZ-5 **f** and CO-MZ-6 **i**. Also note the heterogeneous CK 20 pattern, with major **f** or minor **i** cell populations being immunostained.

tumour. The cell lines OV-MZ-15 and CO-MZ-5 did not give rise to transplant tumours within the observation period. A nude mice inoculation was not performed with the cell line CO-MZ-6.

*Morphological characteristics of the cell lines*

The cell size of the ovarian cancer cells was larger by phase-contrast microscopy and they expanded more diffusely than the colon cancer cell lines, which grew in compact colonies. Transmission electron microscopy revealed only minor differences between the ovarian (Figure 1a–c) and colonic (Figure 1d–f) carcinoma cell lines. The tumour cells were separated by narrow intercellular spaces bridged by cytoplasmic interdigitations and typical desmosomes (Figure 1c and f). Occasionally, the tumour cells exhibited intracellular lumina, indicating their origin from gland-forming adenocarcinomas. The cytoplasm of the cells was rich in polyribosomes and mitochondria, whereas profiles of rough endoplasmic reticulum were seldom seen. Deposits of monoparticulate glycogen were occasionally observed in ovarian and colon carcinoma cells (Figure 1b and f), the colon carcinoma cells sometimes showing microvillus-like cytoplasmic protrusions (Figure 1e). The nuclei were moderately irregular in shape, often exhibiting prominent nucleoli.

*Patterns of IF proteins*

*Cell lines* Results concerning the expression of individual CK polypeptides and vimentin are summarised in Table II and illustrated in Figure 2. All four cell lines strongly expressed the general simple epithelial CK polypeptides CK 18 and 19, along with generally poor expression of stratified epithelial CKs. Differences were observed with respect to the selective simple epithelial CKs: CK 7 was only expressed in ovarian carcinoma cell lines but was completely absent from the colon carcinoma cell lines. In contrast, CK 20 has been found exclusively in the colon carcinoma cell lines but not in the ovarian carcinoma lines. Vimentin was strongly expressed in the ovarian cell lines but was essentially absent from the colon carcinoma cell lines.

In addition, we examined the expression of the CK polypeptides and vimentin in the three colon cancer cell lines CO-MZ-1, -2 and -4, which were also established by our group (results not shown). As expected, these three colon cancer cell lines were also negative for the expression of CK 7 and vimentin, but were highly positive for the expression of CK 20.

Figure 2 shows the immunocytochemical cytokeratin typing of the ovarian cancer cell line OV-MZ-15 and the two colon cancer cell lines. Positive immunocytochemical staining generally consisted of a distinctly fibrillar cytoplasmic pattern.

*Original tumours* Table III shows the expression of the most important simple epithelial cytokeratins and vimentin in the original tumours. The results are in complete agreement with the immunocytochemical staining of the derived cell lines. Again CK 7 and vimentin were expressed by the original tumours of ovarian origin but were completely absent in colon cancer. In contrast, CK 20 was only found in 4% of cells in one ovarian cancer, but was highly expressed in the original colon cancer. Concerning CO-MZ-5 the original sigmoid cancer and the metastasis of the ovary were both consistently positive in 50% and 45% of the cells respectively.

**Table III** Expression of simple epithelial cytokeratin and vimentin in the original tumours of the established cell lines

| | *Simple epithelial cytokeratins* | | | |
|---|---|---|---|---|
| | *CK 7* | *CK 19* | *CK 20* | *Vimentin* |
| OV-MZ-10 | ++ | +++ | (+) | ++ |
| OV-MZ-15 | +++ | +++ | – | +++ |
| CO-MZ-5 | | | | |
| Sigmoid | – | +++ | ++ | – |
| Ovary | – | +++ | ++ | – |
| CO-MZ-6 | – | +++ | +++ | – |

The proportions of immunostained cells were assessed semiquantitatively and scored as follows: –, negative; (+), <5% of cells positive; +, 5–20% of cells positive; ++, 21–80% of cells positive; +++, >80% positive.

*Determination of CA-125 and CEA*

Table IV shows the expression of the tumour-associated antigens *in vivo* and *in vitro*. In three patients the preoperative serum levels were determined. CA-125 was elevated in 2/3 patients, CEA in 3/3 patients. This was in accordance with the IRS score of the original tumour. The original sigmoid cancer and the metastasis of the ovary of CO-MZ-5 both showed an identical staining.

We also performed a FACScan analysis of the cell lines. The percentage of positive cells varied from 0 to 48% for CA-125 and from 0 to 28% for CEA.

### Discussion

Two human ovarian and two colon carcinoma cell lines have been newly established in permanent cell culture.

By clinical criteria, three patients were treated as ovarian cancer patients and one patient was diagnosed as having simultaneous ovarian and colon cancers. Because the cell line of this patient was established from the ovarian tumour, in the beginning all four lines were classified as ovarian cancer cell lines in our laboratory. The clinical diagnosis was fully confirmed by histopathological examinations. The tumours of the two patients that gave rise to the ovarian cancer cell lines OV-MZ-10 and OV-MZ-15 were correctly described as serous cystadenocarcinomas. The colon cell line CO-MZ-5 was derived from a patient with a tumour of both ovaries and a second tumour of the sigmoid colon. The pathologist described a serous cystadenocarcinoma of both ovaries and a second independent primary ulcerating adenocarcinoma of the colon. The tumour from which the colon cell line CO-MZ-6 was established was originally considered to be a serous cystadenocarcinoma of the ovaries. The clinical course of CO-MZ-5, however, was unusual for ovarian cancer. After a histopathologically confirmed complete remission, the patient died of progressive bone metastasis. The other patient died 8 months after diagnosis despite palliative chemotherapy of progressive intraperitoneal disease, compatible with progressive ovarian cancer.

Transmission electron microscopy showed only minor differences between the ovarian and colon carcinoma cell lines. The epithelial nature of the cell lines was confirmed by the presence of numerous desmosomes and occasional intracellular gland-like spaces. The inability to produce transplant tumours in nude mice, which became evident for the cell lines OV-MZ-15 and CO-MZ-5, did not argue against the carcinomatous derivation of these cell lines, since this phenomenon has also been reported in other human tumour cell lines (Hill *et al.*, 1987). The slow median doubling time of 17 days may have impeded the tumorigenesis of CO-MZ-5.

Only cytokeratin analysis enabled us to unmask the true origin of the two colonic carcinoma cell lines. The consistent expression of the simple epithelial CK polypeptides, CK 18 and CK 19, and the poor expression of stratified epithelial CKs provided support for the epithelial nature and derivation of the four lines and was in accordance with the fact that these cell lines were derived from adenocarcinomas.

The simple epithelial cytokeratin CK 7 was only expressed in ovarian carcinoma cells and was completely absent from colon carcinoma cell lines. This reflects exactly the situation in corresponding *in vivo* carcinomas (Moll *et al.*, 1982; 1983; 1992; Osborn *et al.*, 1986; van Niekerk *et al.*, 1991): colon carcinomas are essentially CK 7 negative (for exceptions, see van Niekerk *et al.*, 1991; Moll *et al.*, 1993); ovarian carcinomas are constantly positive. This was confirmed by our study. The colon cancer cell lines, the corresponding original tumour and the metastasis in the ovary were CK 7 negative, whereas the ovarian cancer cell lines and the original tumours were CK 7 positive. Thus, CK 7 can be used as a good discriminating marker, although it should be noted that occasionally cell lines derived from ovarian carcinoma may lose CK 7 expression (Möbus *et al.*, 1992).

CK 20 has been found exclusively in the colon carcinoma cell lines but not in the ovarian carcinoma lines. This result also completely agreed with the clinical situation in '*in situ*' tumours. CK 20, which only recently has been introduced as a new CK polypeptide (Moll *et al.*, 1990), shows a very restricted tissue specificity and is only expressed at significant levels in the mucosa of small and large intestine, in the gastric foveolar epithelium, in the umbrella cells of the urothelium and in epidermal Merkel cells. This specificity is largely maintained in the corresponding carcinomas: in an extended immunohistochemical screening, 89/92 cases of colorectal adenocarcinomas expressed CK 20, whereas 31 out of 34 serous, endometrioid, anaplastic and clear cell ovarian carcinomas were completely negative and the other three were essentially negative for this CK (Moll *et al.*, 1992). The expression of CK 20 is a very stable feature of normal and malignant intestinal epithelium since it is preserved in most metastases of colorectal carcinomas as well as in most established colon carcinoma cell lines (Moll *et al.*, 1990, 1992; 1993). This statement was fully confirmed by the results of our experiments. The original tumours of the colon cancer cell lines were positive for CK 20, as was the metastasis in the ovary. The two reported colon cancer cell lines in this paper and three more colon cell lines, also established by our group, were without exception also positive for CK 20 and negative for CK 7. The original tumours of the reported ovarian cancer cell lines as well as the cell lines themselves were negative.

The only point to be considered when using CK 20 as a marker for particular pathways of epithelial differentiation, notably of the intestinal type, is the fact that ovarian mucinous tumours (both adenomas and carcinomas) also

**Table IV** Preoperative tumour marker level in the patient serum, IRS score of primary tumour and percentage of FACScan-positive cells in cell culture

| | *Patient's serum level preoperatively* | | *IRS score of primary tumour* | | *FACScan-positive cells (%)* | |
|---|---|---|---|---|---|---|
| *Cell line* | *CA-125 (U ml⁻¹)* | *CEA (ng ml⁻¹)* | *CA-125 (U ml⁻¹)* | *CEA (ng ml⁻¹)* | *CA-125 (U ml⁻¹)* | *CEA (ng ml⁻¹)* |
| OV-MZ-10 | 227 | 10 | 4 | 4 | 32 | 0 |
| OV-MZ-15 | nd | nd | 4 | 1 | 48 | 0 |
| CO-MZ-5 | 5 | 245 | 0 | 6 (ovary) | 0 | 0 |
| | | | 0 | 8 (sigmoid) | | |
| CO-MZ-6 | 199 | 216 | 2 | 12 | 10 | 28 |

ND, not determined.

express this CK (Moll *et al*., 1992). A normal ovarian cell expressing CK 20, however, has not yet been discovered. Ovarian mucinous carcinomas and colorectal adenocarcinomas are similar also with respect to other markers including especially CEA positivity. In the present cases the possibility that the two colon cancer cell lines were established from mucinous ovarian cancer could be excluded on the basis of clinical and histopathological findings and by the critical review of the original histology by two independent pathologists. Thus the different patterns of expression of CK 20 enabled us to definitely identify two supposed ovarian carcinoma cell lines as colon carcinoma lines. Retrospectively the supposed independent ovarian tumour of CO-MZ-5 has to be considered a primary metastasis of the colon cancer. The clinical presentation of CO-MZ-6 was difficult to evaluate. The cul-de-sac was padded by tumour and a barium enema of the colon had not been performed preoperatively. CK analysis demonstrated that this patient's tumour was incorrectly classified as ovarian cancer.

An additional discriminative marker is vimentin, which was strongly expressed in the ovarian carcinoma cell lines but essentially absent from the colon cell lines. This is again in very good agreement with the '*in situ*' situation (Azumi & Battifora, 1987; Moll *et al*., 1991). In previous studies, ovarian cancer cell lines all expressed vimentin, albeit at different levels (Möbus *et al*., 1992).

The four cell lines also differed in their expression of tumour-associated antigens. In contrast to the detection of CK 20, however, the different levels of expression of these markers had no impact on differential diagnosis. It is well known that CA-125 is the leading tumour marker of serous cystadenocarcinoma and undifferentiated carcinoma of the ovary. In advanced disease approximately 80% of the patients have an elevated marker (Soper *et al*., 1990). In contrast, CEA shows elevated serum levels in only 30–40% of ovarian cancer patients, mostly in cases of advanced disease and especially in poorly differentiated or mucinous carcinoma, which was excluded in our cell lines by histopathological examination of the original tumour. In colon cancer CEA is consistently the most important marker.

It is obvious that in the serum of the two colon cancer patients CEA was strongly elevated preoperatively. However, in one of these patients (CO-MZ-6), CA-125 was also significantly elevated, supporting the initial diagnosis of ovarian carcinoma. In the one ovarian cancer cell line tested CA-125 was elevated much more than CEA. The results of the serum values agreed with the IRS score of the primary tumour.

The results of the FACScan analysis for CA-125 were in agreement with the patient's serum level and the IRS score of the original tumour. Concerning CEA, the cells of OV-MZ-10 and CO-MZ-5 were negative by FACScan analysis although the lines were established from patients with preoperatively elevated CEA serum levels and a positive IRS score of the primary tumour. This observation suggests the possibility of a clonal selection of CEA-negative tumour cells under the conditions of long-term *in vitro* culture. An analogous loss of CA-125 expression after prolonged culturing of ovarian carcinoma cell lines has also been described by van Niekerk *et al*. (1988) and by our group (Möbus *et al*., 1992).

Our group has established 40 new ovarian cancer cell lines in the past 8 years. They were all characterized exhaustively, including morphological and CK analysis. CK analysis revealed two cell lines (5%) which were highly positive for the expression of CK 20 and thus could be identified as colon cancer lines. Neither the clinical presentation or histological examination of the original tumour nor the expression of tumour-associated antigens was decisive in the differential diagnosis between colon and ovarian cancer.

Permanent cell lines are valuable tools in the examination of the biological properties of human cancers of different origins. Therefore it is important to define the origin of each cell line as exactly as possible. Although CK analysis is not a universal tool differentiating between all adenocarcinomas, we believe that the examination of any established adenocarcinoma cell line with respect to its cytokeratin expression pattern is warranted to further reduce the hazards of misclassification.

### References

AZUMI, N. & BATTIFORA, H. (1987). The distribution of vimentin and keratin in epithelial and nonepithelial neoplasms: a comprehensive immunohistochemical study on formalin- and alcohol-fixed tumors. *Am. J. Clin. Pathol*., **88**, 286–296.

FANNING, J., BIDDLE, W.C., GOLDROSEN, M., CRICKARD, K., CRICKARD, U., PIVER, M.S. & FOON, K.A. (1990). Comparison of cisplatin and carboplatin cytotoxicity in human ovarian cancer cell lines using the MTT assay. *Gynecol. Oncol*., **39**, 119–122.

FRANKE, W.W. & MOLL, R. (1987). Cytoskeletal components of lymphoid organs. *Differentiation*, **36**, 145–163.

GIANCOTTI, F.R., DORSETT, B.H., WEAVER, S.C., BHARATHUR, R., IOACHIM, H.L. & BARBER, H.R.K. (1989). Description of an endometrioid ovarian cancer cell line. *Gynecol. Oncol*., **35**, 330–337.

HILL, B.T., WHELAN, R.D.H., GIBBY, E.M., SHEER, D., HARKING, L.K., SHELLARD, S.A. & RUPNIAK, H.T. (1987). Establishment and characterization of three new human ovarian carcinoma cell lines and initial evaluation of their potential in experimental chemotherapy studies. *Int. J. Cancer*, **39**, 219–225.

HILLS, C.A., KELLAND, L.R., ABEL, G., SIRACKY, J., WILSON, A.P. & HARRAP, K.R. (1989). Biological properties of ten human ovarian carcinoma cell lines: calibration *in vitro* against four platinum complexes. *Br. J. Cancer*, **59**, 527–534.

LANGDON, S.P., HAWKES, M.M., HAY, F.G., LAWRIE, S.S., SCHOL, D.J., HILGERS, J., LEONARD, R.C.F. & SMYTH, J.F. (1988). Effect of sodium butyrate and other differentiation inducers on poorly differentiated human ovarian adenocarcinoma cell lines. *Cancer Res*., **48**, 6161–6165.

LYNCH, M.H., O'GUIN, W.M., HARDY, C., MAK, L. & SUN, T.-T. (1986). Acidic and basic hair/nail ('hard') keratins: their colocalization in upper cortical and cuticle cells of human hair follicle and their relationship to 'soft' keratins. *J. Cell. Biol*., **103**, 2593–2606.

MIOTTI, S., CANEVARI, S., MENARD, D., MEZZANZANICA, D., PORRO, G., PUPA, S.M., REGAZZONI, M., TAGLIABUE, E. & COLNAGHI, M.J. (1987). Characterization of human ovarian carcinoma-associated antigens defined by novel monoclonal antibodies with tumor-restricted specificity. *Int. J. Cancer*, **39**, 297–303.

MÖBUS, V.J., GERHARZ, C.D., PRESS, U., MOLL, R., BECK, T., MELLIN, W., POLLOW, K., KNAPSTEIN, P.G. & KREIENBERG, R. (1992). Morphological, immunohistochemical and biochemical characterization of 6 newly established human ovarian carcinoma cell lines. *Int. J. Cancer*, **52**, 76–84.

MOLL, R., FRANKE, W.W., SCHILLER, D.L., GEIGER, B. & KREPLER, R. (1982). The catalog of human cytokeratins: patterns of expression of specific cytokeratins in normal epithelia, tumors, and cultured cells. *Cell*, **31**, 11–24.

MOLL, R., KREPLER, R. & FRANKE, W.W. (1983). Complex cytokeratin polypeptide patterns observed in certain human carcinomas. *Differentiation*, **23**, 256–269.

MOLL, R., SCHILLER, D.L. & FRANKE, W.W. (1990). Identification of protein II of the intestinal cytoskeleton as a novel type-I cytokeratin with unusual properties and expression patterns. *J. Cell Biol*., **111**, 567–580.

MOLL, R., PITZ, S., LEVY, R., WEIKEL, W., FRANKE, W.W. & CZERNOBILSKY, B. (1991). Complexity of expression of intermediate filament proteins, including glial filament protein, in endometrial and ovarian adenocarcinomas. *Hum. Pathol*., **22**, 989–1001.

MOLL, R., LÖWE, A., LAUFER, J. & FRANKE, W.W. (1992). Cytokeratin 20 in human carcinomas. A new histodiagnostic marker detected by monoclonal antibodies. *Am. J. Pathol*., **140**, 427–447.

MOLL, R., ZIMBELMANN, R., GOLDSCHMIDT, M.D., KEITH, M., LAUFER, J., KASPER, M., KOCH, P.J. & FRANKE, W.W. (1993). The human gene encoding cytokeratin 20 and its expression during fetal development and in gastrointestinal carcinomas. *Differentiation* (in press).

NIEKERK, C.C., VAN, POELS, L.G., JAP, P.H.K., SMEETS, D.F.C.M., THOMAS, C.M.G., RAMAEKERS, F.C.S. & VOOIJS, G.P. (1988). Characterization of a human ovarian carcinoma cell line, OTN 14, derived from a mucinous cystadenocarcinoma. *Int. J. Cancer*, **42**, 104–111.

NIEKERK, C.C., VAN, JAP, P.H.K., RAMAEKERS, F.C.S., MOLENGRAFT, F. & VAN DE POELS, L.G. (1991). Immunohistochemical demonstration of keratin 7 in routinely fixed paraffin-embedded human tissues. *J. Pathol.*, **165**, 145–152.

OSBORN, M., LESSEN, G., VAN, WEBER, K., KLÖPPEL, G., ALTMANNSBERGER, M. (1986). Differential diagnosis of gastrointestinal carcinomas by using monoclonal antibodies specific for individual keratin polypeptides. *Lab. Invest.*, **55**, 497–504.

PITZ, S., MOLL, R., STÖRKEL, S. & THOENES, W. (1987). Expression of intermediate filament proteins in sub-types of renal-cell carcinomas and in renal oncocytomas. Distinction of two classes of renal-cell tumors. *Lab. Invest.*, **56**, 642–653.

RUTZKY, L.P., TOMITA, J.T., CALENOFF, M.A. & KAHAN, B.D. (1979). Human colon adenocarcinoma cells. III. *In vitro* organoid expression and carcinoembryonic antigen kinetics in hollow fiber culture. *J. Natl Cancer Inst.*, **63**, 893–899.

SHI, Z.R., TSAO, D. & KIM, Y.S. (1983). Subcellular distribution, synthesis and release of carcinoembryonic antigen in cultured human colon adenocarcinoma cell lines. *Cancer Res.*, **43**, 4045–4049.

SOPER, J.T., HUNTER, V.J., DALY, L., TANNER, M., CREASMAN, W.T. & BAST, R.C. (1990). Pre-operative serum tumor-associated antigen levels in women with pelvic masses. *Obstet. Gynecol.*, **75**, 249–254.

TROYANOVSKY, S.M. & GUELSTEIN, V.I., TCHIPYSHEVA, T.A., KRUTOVSKIKH, V.A. & BANNIKOV, G.A. (1989). Patterns of expression of keratin 17 in human epithelia: dependency on cell position. *J. Cell Sci.*, **93**, 419–426.

WOLF, C.W., HAYWARD, J.P., LAWRIE, S.S., BUCKTON, K., MCINTYRE, M.A., ADAMS, D.J., LEWIS, A.D., SCOTT, A.R.R. & SMYTH, J.F. (1987). Cellular heterogeneity and drug resistance in two ovarian adenocarcinoma cell lines derived from a single patient. *Int. J. Cancer*, **39**, 695–702.